# The association of depressive symptoms with cardiovascular and all-cause mortality in Central and Eastern Europe: Prospective results of the HAPIEE study

**DOI:** 10.1177/2047487316649493

**Published:** 2016-05-06

**Authors:** Magdalena Kozela, Martin Bobak, Agnieszka Besala, Agnieszka Micek, Ruzena Kubinova, Sofia Malyutina, Diana Denisova, Marcus Richards, Hynek Pikhart, Anne Peasey, Michael Marmot, Andrzej Pająk

**Affiliations:** 1 Department of Epidemiology and Population Studies, Jagiellonian University Medical College, Krakow, Poland; 2 Department of Epidemiology and Public Health, University College London, London, UK; 3 National Institute of Public Health, Prague, Czech Republic; 4 Institute of Internal and Preventive Medicine, Novosibirsk, Russia; 5 Novosibirsk State Medical University, Novosibirsk, Russia; 6 MRC Unit for Lifelong Health and Ageing, University College London, London, UK

**Keywords:** Depressive symptoms, mortality, Eastern Europe, cardiovascular disease

## Abstract

**Background:**

Studies in western populations have shown a positive association between depression and cardiovascular disease (CVD) and all-cause mortality. The association with depressive symptoms seems to be graded, rather than limited to the presence versus the absence of depression. Evidence from populations with potentially different patterns of confounders helps to address the consistency of these findings. The objective of the study was to investigate the association between depressive symptoms and all-cause and CVD mortality in populations of Central and Eastern Europe.

**Study design:**

This was a prospective cohort study.

**Methods:**

A total of 24,542 participants aged 45–69 years, randomly selected from populations of Novosibirsk (Russia), Krakow (Poland) and six Czech towns, were included. Depressive symptoms, assessed by the 20-item Center for Epidemiologic Studies Depression (CES-D) scale, were used as both continuous and categorical variables. Data on deaths were obtained from local or national death registers. Associations between depression and mortality were assessed using Cox proportional hazards models.

**Results:**

Over a median of 7 years, 2091 deaths from all causes and 850 CVD deaths occurred in the cohorts. There was a graded association between CES-D score and mortality; the hazard ratio (HR) of CVD mortality for a 1 SD increase in CES-D was 1.20 (95% confidence interval (CI): 1.16–1.24) in men and 1.23 (95% CI: 1.12–1.35) in women; for all-cause mortality, the HRs were 1.13 (95% CI: 1.09–1.18) and 1.17 (95% CI: 1.10–1.25), respectively. The results were similar across countries.

**Conclusions:**

Depressive symptoms predicted CVD and all-cause mortality independently of a wide range of potential confounders. The association followed a gradient and increased mortality risks were associated with scores below the cut-offs that are commonly used to define ‘depression’.

## Introduction

Numerous studies have shown that depression increases the risk of cardiovascular disease (CVD), largely independently of other risk factors.^1–3^ Several mechanisms are suggested to explain this association, including health behaviours (e.g. smoking, physical inactivity or alcohol consumption) and direct effects (e.g. via increased platelet activity, inﬂammation, alterations in the hypothalamic–pituitary–adrenal axis or increased activity of the sympathetic nervous system).^4–8^ However, the causal role of depression remains debated because the observational evidence is not entirely consistent^9^ and because experimental studies of the effects of depression treatment on cardiovascular outcomes produced ambiguous results.^10–13^ Another question relates to whether mortality is associated only with severe depression or whether it is also predicted by milder symptoms of psychological distress.^14^

As long as studies of the relationship between depression and CVD adopt observational designs, there will be problems of potential confounding. One way to address this issue is to conduct studies in different settings. In different social contexts, the correlation between depression, confounding factors and CVD may follow different patterns; this may help establish whether the observational associations are due to confounding. In Central and Eastern Europe, the structure and correlation of potential confounders differ from Western countries, where the vast majority of previous observational studies have been based. In addition, Central and Eastern European countries have undergone rapid and radical political, economic and social changes. It is not implausible that these changes affected mental well-being, and it has been estimated that the burden of ill health attributable to major depression was five-times higher in Eastern Europe than in Western Europe.^15^ Confirmation of this association in different social contexts provides the evidence that is necessary to assess whether the observed association is causal and whether psychological distress or depression can be regarded as cardiovascular risk factors.

The objective of the present study was to investigate the association between depressive symptoms and all-cause and CVD mortality in populations of Central and Eastern Europe and to assess whether the association between depressive symptoms and CVD is graded and is present across the full range of depressive symptoms severity.

## Methods

### Study population and subjects

The Health, Alcohol and Psychosocial factors In Eastern Europe (HAPIEE) project recruited random population samples in Krakow (Poland), Novosibirsk (Russia) and six Czech towns (Havirov-Karvina, Hradec Kralove, Jihlava, Kromeriz, Liberec and Usti nad Labem). Comprehensive information on the methodology of the HAPIEE project was previously described.^16^ A summary of information that is important for this paper is given below.

The baseline examination was conducted between 2002 and 2005 in a total number of 28,945 men and women aged 45–69 years (overall participation rate: 59%).^16^ All participants gave written consent.

### Questionnaires

At baseline, participants were interviewed by trained nurses using a standardized questionnaire. In the HAPIEE project, all questionnaires were translated from English into each language and back translated into English to check for accuracy. Information on depressive symptoms, age, education, marital status, employment, health status (history of CVD, cancer, hypertension, diabetes or hypercholesterolaemia) and health behaviours (smoking, physical activity and alcohol intake) was obtained.

Depressive symptoms were assessed using the Center for Epidemiologic Studies Depression (CES-D) scale.^17^ This consisted of 20 self-reported items referring to symptoms occurring during the past week. The severity of each item was scored 0, 1, 2 or 3; thus, the total score range was 0–60. Calculation of the final score allowed for a maximum of four missing answers; in those who gave between 16 and 19 responses, the average score from the answers was calculated, divided by the number of questions answered and multiplied by 20. The CES-D scale was previously validated in all three countries.^18–20^ In the analysis, the depression score was used as (1) a continuous variable and (2) a variable with four categories: free of depression symptoms (0–10), low (11–15), mild (16–19) and moderate/severe depressive symptoms (20–60). The original CES-D tool suggested that a score ≥16 had good sensitivity and specificity^21^; the remaining cut-offs were adopted arbitrarily in order to ensure sufficient numbers of subjects per category.

Physical activity was assessed by a question about duration of leisure time and sport-related physical activity in a typical week. Current smoking status was assigned for persons smoking cigarettes regularly or occasionally. Ex-smokers were those who had smoked in the past but stopped. Persons who had never smoked cigarettes were considered to be non-smokers. Alcohol intake was assessed using the Graduated Frequency Questionnaire.^22^ History of doctor-diagnosed CVD and cancer with and without hospitalisation were coded as dichotomous variables.

### Physical examination

Height and weight were measured in the vertical position using scales with a built-in ruler, with participants wearing light indoor clothing and no shoes. Body mass index (BMI) was calculated in terms of kg/m^2^. Blood pressure was measured after at least a 5-minute rest, in a sitting position, on the right arm, three times at two-minute intervals using an Omron M5-I digital blood pressure monitor. The average of the last two measurements was used in the analysis. Hypertension was defined as blood pressure ≥140/90 mmHg or receiving antihypertensive treatment in the 2 weeks prior to examination.

### Blood tests

Venous blood was collected using vacuum tubes from participants after overnight fasting. Blood was stored at +4℃ and centrifuged within 4 hours of the venepuncture. In Poland, the analyses were carried out on the same day; in the Czech Republic and Russia, serum samples were frozen and stored at –20℃ until the analysis. In all centres, blood lipids were determined by the automated enzymatic colorimetric method^23^ using reagents from Boehringer Mannheim Diagnostics and Hoffmann-La Roche. Low-density lipoprotein cholesterol was calculated by the Friedewald formula. Hypercholesterolaemia was defined as total cholesterol ≥5 mmol/L, low-density lipoprotein cholesterol ≥3 mmol/L or receiving lipid-lowering treatment. Glucose concentration in plasma was assessed using the enzymatic method. Diabetes was defined as having fasting plasma glucose ≥7 mmol/L or having diabetes diagnosed by a doctor.

### Registration of deaths

The cohorts were followed up for cause-specific mortality until 31 December 2010 in Poland and Russia and until 31 December 2011 in the Czech Republic. In Russia, information on deaths was obtained from the death register developed by the Institute of Internal Medicine, based on data from medical death certificates, the Novosibirsk office of the State Statistical Bureau (Goscomstat) and from the population registration bureau. In Poland, data from the Local Register of Residents of the City of Krakow and Central Statistical Office were used. In the Czech Republic, data from the National Death Register were used. Cardiovascular deaths were those with International Statistical Classification of Diseases and Related Health Problems (10th edition) (ICD-10) codes I.00–I.99. The mortality register in Novosibirsk was set up for the World Health Organization MONItoring trends and determinants in CArdiovascular disease (MONICA) project^24^ and has been in operation since then; it is believed to provide complete coverage of deaths in the study population.^25,26^ Both Poland and the Czech Republic are countries with nearly 100% completeness of the registration of death and good accuracy of certification of the causes of deaths for the broad category ‘diseases of the circulatory system’ (ICD-10: I.00–I.99).^27,28^

### Statistical analyses

Participants who agreed to follow-up and had non-missing data on CES-D were included in the analyses. A total of 2,452 Russian participants who were interviewed by nurses and whose data failed quality control for CES-D were excluded from the analysis.^29^ The distributions of CES-D scores and other variables in men and women were examined in each country and in the whole study sample. The relationships between CES-D score and all-cause and CVD mortality were assessed using the Cox proportional hazards model with two levels of adjustment: (1) adjustment for age (continuous); and (2) adjustment for age, education (categorical), marital status (binary), occupational status (binary), history of CVD (binary), smoking, (categorical), BMI (continuous), hypercholesterolaemia (binary), hypertension (binary), alcohol intake (continuous), physical activity (continuous) and history of cancer (binary) in analyses of all-cause mortality. Proportional hazard assumptions were checked using Schoenfeld residuals. The timescale used was the time since assessment of exposure (alternative use of age as a timescale produced virtually identical results). Because of the two-stage nature of the examination, the participation rate for the clinical examination was lower than for the interview. Thus, the number of persons included in the multivariate models was lower (by approximately 16%), as the sample was restricted to participants without missing data on any of the covariates (9575 men and 10,920 women). Interactions between depression symptoms and country, sex and other variables were assessed using likelihood ratio tests. Since there were no significant interactions (all p-values >0.1), the data from the three countries were pooled. Models were fitted with CES-D score, used as both a continuous and a categorical variable. In the pooled sample, using all three countries together, clustered analysis with robust standard errors was performed. All statistical analyses were conducted using Stata version 12.1 (StataCorp LP, TX, USA).

## Results

There were 13,617 men and 15,328 women examined at baseline (response rate: 59%). Out of these, 11,528 men and 13,014 women agreed to follow-up and had non-missing data on CES-D. In total, there were 80,517 and 94,316 person-years of observation in men and women, respectively; among these, 2091 participants died during the follow-up, and CVD was the most common cause of deaths both in men (41%) and women (39%) (Table 1).

**Table 1.** Sample size and number of deaths by country and sex.

	Czech Republic		Russia		Poland		Total	
	*n*	%	*n*	%	*n*	%	*n*	%
*Men*								
Baseline examination	4123		4264		5230		13,617	
Follow-up	3955	95.9	4190	98.3	4872	93.2	13,017	95.6
Follow-up + CES-D	3707	89.9	3046	71.4	4775	91.3	11,528	84.7
Person-years	29,193		18,433		32,892		80,517	
Number of deaths	431	11.6	431	14.3	528	11.1	1390	12.1
Number of CVD deaths	162	4.4	230	7.6	184	3.9	576	5.0
*Women*								
Baseline examination	4734		5096		5498		15,328	
Follow-up	4517	95.4	5028	98.7	5140	93.5	14,685	95.8
Follow-up + CES-D	4230	89.4	3748	73.5	5036	91.6	13,014	84.9
Person-years	34,254		24,324		35,739		94,316	
Number of deaths	232	5.5	185	5.0	284	5.6	701	5.4
Number of CVD deaths	76	1.8	102	2.7	96	1.9	274	2.1

CES-D: Center for Epidemiologic Studies Depression; CVD: cardiovascular disease.

The distribution of the baseline CES-D score was highly right-skewed in all countries. A higher proportion of men than women were free of depressive symptoms (63% vs. 48%) (Supplementary Figure 1).

The mean age at baseline was 58.2 years (SD = 7.0) in men and 57.7 years (SD = 7.1) in women (Supplementary Tables 1 and 2). A high percentage of participants had secondary education. In all countries, more men than women were married or cohabiting and professionally active. Smoking patterns in men and women were similar in Poland and in the Czech Republic, while in Russia, the proportion of smokers was very high in men and very low in women. In all cohorts, alcohol consumption was higher in men. Hypertension and diabetes were more prevalent in men, while hypercholesterolaemia was more frequent in women. A positive history of CVD was reported by 21% of men and 18% of women.

The associations between depressive symptoms and CVD mortality are shown in Table 2. After adjustment for age, the risk of CVD mortality in the highest category of CES-D score was approximately double that of the lowest category. Pooled analysis of all three countries confirmed a graded increase in CVD mortality by depressive symptoms in both sexes. Further adjustment for education, occupational status, smoking, BMI, hypercholesterolaemia, hypertension, alcohol intake and history of CVD attenuated the relationship by approximately 40%. Nevertheless, the graded associations remained significant; in analyses of the continuous CES-D score, the adjusted hazard ratio (HR) for a 1-SD increase in score was 1.20 (95% confidence interval (CI): 1.16–1.24) in men and 1.23 (95% CI: 1.12–1.35) in women.

**Table 2.** Association between Center for Epidemiologic Studies Depression scores and risk of cardiovascular disease death by sex.

		Czech Republic	Russia	Poland	All three countries (cluster)	*p*-value for
CES-D score		HR (95% CI)	HR (95% CI)	HR (95% CI)	HR (95% CI)	interaction
*Men*						
Age-adjusted	0–10	1.00	1.00	1.00	1.00	
	11–15	1.30 (0.89–1.89)	1.30 (0.94–1.80)	1.38 (0.93–2.03)	1.32 (1.28-1.36)	
	16–20	1.24 (0.68–2.26)	1.91 (1.26–2.88)	2.13 (1.39–3.27)	1.78 (1.52–2.08)	0.805
	21+	2.34 (1.43–3.83)	2.14 (1.43–3.22)	2.72 (1.83–4.03)	2.36 (2.09–2.66)	
	*p*-value for trend	0.002	<0.001	<0.001	<0.001	
	1 SD increase	1.30 (1.14–1.47)	1.29 (1.16–1.44)	1.43 (1.27–1.61)	1.35 (0–1.31)	0.574
Fully adjusted^a^	0–10	1.00	1.00	1.00	1.00	
	11–15	0.85 (0.52–1.39)	1.21 (0.87–1.69)	1.00 (0.62–1.61)	1.03 (0.86–1.23)	
	16–20	1.17 (0.59–2.31)	1.63 (1.07–2.48)	1.31 (0.76–2.27)	1.37 (1.23–1.54)	0.949
	21+	1.82 (0.98–3.36)	1.63 (1.07–2.49)	1.76 (1.07–2.88)	1.68 (1.62–1.74)	
	*p*-value for trend	0.130	0.005	0.033	<0.001	
	1 SD increase	1.18 (1.00–1.40)	1.19 (1.06–1.34)	1.2 (1.01–1.42)	1.20 (1.16–1.24)	0.972
*Women*						
Age-adjusted	0–10	1.00	1.00	1.00	1.00	
	11–15	1.72 (0.98–3.03)	1.37 (0.81–2.34)	1.54 (0.90–2.63)	1.57 (1.43–1.74)	
	16–20	1.58 (0.75–3.36)	1.51 (0.85–2.68)	1.67 (0.93–2.97)	1.71 (1.63–1.79)	0.992
	21+	2.46 (1.36–4.45)	1.87 (1.12–3.12)	1.86 (1.09–3.18)	2.16 (1.86–2.52)	
	*p*-value for trend	0.003	0.015	0.016	<0.001	
	1 SD increase	1.41 (1.18–1.69)	1.22 (1.02–1.45)	1.32 (1.10–1.58)	1.35 (1.27–1.43)	0.582
Fully adjusted^a^	0–10	1.00	1.00	1.00	1.00	
	11–15	2.30 (1.01–5.27)	1.30 (0.76–2.22)	1.34 (0.65–2.73)	1.60 (1.24–2.06)	
	16–20	2.89 (1.10–7.62)	1.32 (0.74–2.34)	2.19 (1.12–4.30)	2.06 (1.4–3.04)	0.608
	21+	2.15 (0.84–5.47)	1.24 (0.73–2.10)	1.45 (0.68–3.09)	1.77 (1.49–2.11)	
	*p-*value for trend	0.059	0.460	0.150	0.012	
	1 SD increase	1.37 (1.06–1.77)	1.04 (0.86–1.25)	1.18 (0.92–1.52)	1.23 (1.12–1.35)	0.215

a Adjusted for age, education, marital status, occupational status, history of cardiovascular disease, smoking, body mass index, hypercholesterolaemia, hypertension, physical activity and alcohol intake.

CI: confidence interval; CES-D: Center for Epidemiologic Studies Depression; HR: hazard ratio.

The associations of depressive symptoms with all-cause mortality (Table 3) were somewhat weaker than with CVD deaths. In the pooled analysis of the three cohorts, the age-adjusted HRs of death in men and women with CES-D scores of >20 versus ≤10 were 1.94 (95% CI: 1.77–2.13) and 2.14 (95% CI: 1.75–2.61), respectively. Multivariable adjustment reduced the HRs to a greater extent than in analyses of CVD mortality, but in both sexes, the associations of the continuous CES-D score with all-cause mortality remained statistically significant.

**Table 3.** Association between Center for Epidemiologic Studies Depression scores and risk of death from all causes by sex.

		Czech Republic	Russia	Poland	All three countries (cluster)	*p*-value for
CES-D score		HR (95% CI)	HR (95% CI)	HR (95% CI)	HR (95% CI)	interaction
*Men*						
Age-adjusted	0–10	1.00	1.00	1.00	1.00	0.987
	11–15	1.37 (1.09–1.72)	1.42 (1.13–1.79)	1.31 (1.05–1.63)	1.36 (1.31–1.41)	
	16–20	1.79 (1.3–2.46)	1.66 (1.21–2.28)	1.82 (1.41–2.35)	1.76 (1.64–1.88)	
	21+	2.07 (1.5–2.85)	1.84 (1.34–2.52)	1.98 (1.54–2.55)	1.94 (1.77–2.13)	
	*p*-value for trend	<0.001	<0.001	<0.001	<0.001	
	1 SD increase	1.26 (1.17–1.37)	1.21 (1.12–1.31)	1.30 (1.21–1.41)	1.27 (1.24–1.29)	0.785
Fully adjusted^a^	0–10	1.00	1.00	1.00	1.00	
	11–15	1.10 (0.82–1.47)	1.29 (1.02–1.63)	1.10 (0.84–1.44)	1.14 (1.03–1.26)	
	16–20	1.59 (1.07–2.37)	1.44 (1.04–1.99)	1.31 (0.95–1.81)	1.38 (1.22–1.55)	0.773
	21+	1.76 (1.16–2.66)	1.36 (0.97–1.89)	1.31 (0.95–1.8)	1.41 (1.2–1.64)	
	*p*-value for trend	0.002	0.009	0.047	<0.001	
	1 SD increase	1.18 (1.06–1.31)	1.10 (1.01–1.21)	1.12 (1.01–1.24)	1.13 (1.09–1.18)	0.546
*Women*						
Age-adjusted	0–10	1.00	1.00	1.00	1.00	
	11–15	1.59 (1.14–2.21)	1.40 (0.95–2.06)	1.44 (1.05–1.98)	1.49 (1.39–1.6)	
	16–20	1.64 (1.07–2.51)	1.62 (1.07–2.45)	1.49 (1.05–2.12)	1.63 (1.5–1.76)	0.662
	21+	2.54 (1.82–3.54)	1.58 (1.06–2.34)	2.12 (1.58–2.86)	2.14 (1.75–2.61)	
	*p*-value for trend	<0.001	0.017	<0.001	<0.001	
	1 SD increase	1.41 (1.27–1.56)	1.19 (1.04–1.35)	1.34 (1.2–1.48)	1.35 (1.25–1.42)	0.189
Fully adjusted^a^	0–10	1.00	1.00	1.00	1.00	
	11–15	1.28 (0.83–2.00)	1.35 (0.92–1.99)	1.15 (0.79–1.68)	1.30 (1.18–1.44)	
	16–20	1.56 (0.90–2.71)	1.49 (0.99–2.26)	1.18 (0.78–1.79)	1.46 (1.23–1.73)	0.909
	21+	1.61 (1.01–2.6)	1.27 (0.85–1.90)	1.27 (0.86–1.88)	1.47 (1.3–1.65)	
	*p*-value for trend	0.030	0.230	0.210	0.005	
	1 SD increase	1.26 (1.09–1.47)	1.09 (0.95–1.24)	1.10 (0.96–1.26)	1.17 (1.10–1.25)	0.289

a Adjusted for age, education, marital status, occupational status, history of cardiovascular disease, history of cancer, smoking, body mass index, hypercholesterolaemia, hypertension, physical activity and alcohol intake.

CI: confidence interval; CES-D: Center for Epidemiologic Studies Depression; HR: hazard ratio.

Additional sensitivity analyses supported these results. First, exclusion of deaths in first 2 years of follow-up did not change the findings (Supplementary Table 3). Second, the results were unchanged after exclusion of participants who reported existing CVD at baseline (Supplementary Tables 4 and 5). Third, as the covariates in the final model are a mix of potential confounders and mediators, we have estimated models separately adjusted for age plus socioeconomic variables (unlikely to be mediators) and additionally adjusted for potential mediators (e.g. alcohol, smoking and hypertension); there was some attenuation of HRs after adding potential mediators (Supplementary Tables 4 and 5). Finally, the association with non-CVD deaths was weaker than with CVD deaths, confirming a relative specificity of the association with CVD; for example, the fully adjusted HRs for men and women with CES-D scores of >20 versus ≤10 in the combined dataset were 1.94 (95% CI: 1.64–2.29) and 2.1 (95% CI: 1.73–2.55), respectively (data not shown).

## Discussion

This large prospective cohort study found significant positive associations between increasing depressive symptoms and CVD mortality and, to a lesser extent, with all-cause mortality. Although the age-adjusted associations were attenuated after multivariable adjustment, a graded relationship between CES-D score and mortality remained significant. To the best of our knowledge, this is by far the largest prospective cohort study using standard methods in the general population in Central and Eastern Europe.

Several limitations of the study need to be considered. First, the participation rate of 59% may have affected the representativeness of the study sample. Response rates in epidemiological studies have been declining over recent decades, and lower response rates may affect the estimates of disease rates. The analysis of the Polish cohort, in which data on the mortality of both respondents and non-respondents were available, showed that non-respondents had higher mortality and a greater prevalence of CVD risk factors than study participants.^30^ However, empirical data suggest that response rates do not necessarily influence the estimates of the examined associations substantially.^31,32^ At least in Poland, the cohort has been shown to be similar to the general urban population of that country with respect to marital status, occupational status, smoking, BMI, prevalence of hypercholesterolaemia, hypertension and hyperglycaemia and the Systematic COronary Risk Evaluation (SCORE) risk category.^33^ In addition, participants who did not attend the physical examination had a higher mortality risk than those who did attend the physical examination. Therefore, our results are based on the healthier part of the cohort,^30^ and this might have resulted in some underestimation of the associations between depression and mortality.

Second, while the CES-D scale is a widely accepted tool that has been validated in many countries, including those represented in our study, the possibility that the scale’s performance is not completely identical in all populations cannot be excluded. In addition, depressive symptoms were only assessed once (at baseline) and we were not able to consider the course of depression. One would expect that that these issues would underestimate the associations with mortality.

Third, a major issue in observational studies of depression and mortality is the independence of the observed association. Depression and distress are associated with numerous factors (e.g. socioeconomic status and health behaviours), which may confound or mediate the association.^34^ Although we adjusted for a large number of such factors, it cannot be certain whether the remaining effect is due to depressive symptoms alone or whether it is due to residual confounding. The substantial reduction of HRs after adjustment may indicate the presence of residual confounding due to unmeasured factors; on the other hand, the multivariable model may also have led to an over-adjustment, since some of the variables included in the final model may be mediators, rather than confounders, or both confounders and mediators (e.g. alcohol). The (relatively modest) attenuation of HRs after the inclusion of potential mediators suggests that the full models may lead to an underestimation of the real effect.

Our findings were generally consistent across the sexes and cohorts. Additional analysis showed no substantial changes in the results after excluding participants with a history of CVD or excluding deaths within the first 2 years of observation; this argues against a major reverse causation bias. Importantly, our results are consistent with most previous studies, confirming depression as a predictor (whether as a causal factor or not) of all-cause and CVD mortality.^2,35,36^ Although the possibility of false-positive findings cannot be excluded, the presence of a graded association, including less severe symptoms, further supports the existence of a genuine association between depression symptoms and mortality.

The causality of this association remains unclear, although there are several psychobiological mechanisms that may plausibly link depression with somatic disorders such as CVD. Depression contributes to hypothalamic–pituitary–adrenal axis hyperactivity. Findings from other studies show that depressed persons are more likely to have increased cortisol and adrenocorticotropic hormone levels, with no effect on corticotropin-releasing hormone.^37^ Dysfunction of the autonomic nervous system may also link depression with CVD, as depression is associated with reduced heart rate variability, which decreases with increasing depression severity.^38^ Furthermore, depressed persons were reported to have higher concentrations of inflammatory biomarkers compared to healthy subjects, and inflammation is an established accelerator of atherosclerosis.^39–41^ Inflammatory responses may be caused by alterations in the autonomic system and in the hypothalamic–pituitary–adrenal axis.^42,43^

On the other hand, intervention studies found that treatment of depression – either pharmacological or behavioural – was associated with modest improvements in depressive symptoms, but were inconclusive in terms of improvements in cardiac outcomes. However, these studies were conducted on patients with existing CVD, and there is no relevant evidence from general population samples.^10,12,13^ The relationship between depression and cardiac outcome could also be confounded by the association between the use of some antidepressants and a higher prevalence of ischaemic heart disease.^44,45^

Whether causal or not, there is solid evidence that depression remains a strong predictor of CVD mortality, with the strength of the association being comparable to classic CVD risk factors. Our study reports the presence of this association in the context of populations undergoing radical societal transformation.^46^ The graded association with symptom severity suggests that the conventional cut-offs for the CES-D scale do not adequately identify persons who are at increased risk of CVD death.

## Supplementary Material

Supplementary material
