# Supplementary material for: The association of depressive symptoms with cardiovascular and all-cause mortality in Central and Eastern Europe: Prospective results of the HAPIEE study
Source: Eur J Prev Cardiol. 2016 May 6;23(17):1839–47. doi: 10.1177/2047487316649493 (PMC5089224; doi:10.1177/2047487316649493)

Supplementary table 1. Distribution of covariates men by country

	**Czech Republic**		**Russia**		**Poland**		**p**	**Total**	
Men									
Age (x,sd)	58.48	7.17	58.11	7.03	57.92	6.95	0.00134	58.15	7.04
Education (n,%)									
primary	211	5.7	179	5.9	442	9.3	<0.0001	832	7.2
vocational	1612	43.7	667	21.9	1304	27.3	<0.0001	3583	31.1
secondary	1182	32	1160	38.1	1594	33.4	<0.0001	3936	34.2
university	685	18.6	1040	34.1	1432	30	<0.0001	3157	27.4
Marital status (n,%)									
married/cohabiting	3098	83.9	2698	88.6	4129	86.7	<0.0001	9925	86.3
single/divorced/widowed	594	16.1	348	11.4	632	13.3	<0.0001	1574	13.7
Occupational status (n,%)	1403	38.2	825	27.1	1722	36.2	<0.0001	3950	34.4
working	2142	58.3	1915	62.9	2303	48.3	<0.0001	6360	55.4
not working	1532	41.7	1131	37.1	2464	51.7	<0.0001	5127	44.6
Smoking (n,%)									
current smoker	1093	29.7	1478	48.5	1715	36	<0.0001	4286	37.3
ex smoker	1403	38.2	825	27.1	1722	36.2	<0.0001	3950	34.4
never smoker	1178	32.1	743	24.4	1324	27.8	<0.0001	3245	28.3
BMI (x,sd)	28.31	3.96	26.72	4.46	28.01	4.04	<0.0001	27.72	4.2
Physical activity (Me,IR)	2	0-6	0	0-4	4	0-8	<0.0001	2	0-7
Alcohol consumption (Me,IR)	3120	545-10150	2570	402.5-7800	800	40-3140	<0.0001	1800	270-6107.5
Hypercholesterolemia (n,%)	2499	78.3	2575	84.6	3617	81.9	<0.0001	8691	81.6
Hypertension (n,%)	2240	72.7	1881	61.8	2784	66.5	<0.0001	6905	66.9
Diabetes (n,%)	736	19.9	333	11.3	772	16.2	<0.0001	1841	16.1
History of CVD (n,%)	566	15.8	737	24.2	1113	23.6	<0.0001	2416	21.3
History of cancer (n,%)	153	4.2	41	1.3	157	3.3	<0.0001	351	3.1

Supplementary table 2. Distribution of covariates in women by country

	Czech Republic		Russia		Poland		p	Total	
Women									
Age (x,sd)	57.8	7.09	57.91	7.1	57.37	6.99	0.00047	57.67	7.06
Education (n,%)									
primary	752	17.8	220	5.9	675	13.4	<0.0001	1647	12.7
vocational	1291	30.6	1136	30.3	762	15.1	<0.0001	3189	24.5
secondary	1738	41.2	1350	36	2232	44.4	<0.0001	5320	40.9
university	437	10.4	1042	27.8	1363	27.1	<0.0001	2842	21.9
Marital status (n,%)									
married/cohabiting	2885	68.4	2277	60.8	3363	66.9	<0.0001	8525	65.6
single/divorced/widowed	1330	31.6	1471	39.2	1664	33.1	<0.0001	4465	34.4
Occupational status (n,%)	933	22.2	185	4.9	1049	20.9	<0.0001	2167	16.7
working	2004	47.6	1779	47.5	1982	39.4	<0.0001	5765	44.4
not working	2204	52.4	1969	52.5	3045	60.6	<0.0001	7218	55.6
Smoking (n,%)									
current smoker	991	23.6	384	10.2	1440	28.7	<0.0001	2815	21.7
ex smoker	933	22.2	185	4.9	1049	20.9	<0.0001	2167	16.7
never smoker	2272	54.1	3179	84.8	2535	50.5	<0.0001	7986	61.6
BMI (x,sd)	28.12	5.06	30.14	5.65	28.38	5.08	<0.0001	28.86	5.34
Physical activity (Me,IR)	2	0-7	0	0-3	4	0-7	<0.0001	2	0-7
Alcohol consumption (Me,IR)	300	40-1480	270	60-480	40	0-360	<0.0001	120	0-660
Hypercholesterolemia (n,%)	3018	81.4	3430	91.9	3974	85.1	<0.0001	10422	86
Hypertension (n,%)	2111	58	2493	66.6	2469	55.8	<0.0001	7073	59.9
Diabetes (n,%)	588	13.9	426	11.7	584	11.6	0.00109	1598	12.4
History of CVD (n,%)	385	9.6	805	21.5	1047	21	<0.0001	2237	17.5
History of cancer (n,%)	335	8.2	158	4.2	311	6.2	<0.0001	804	6.3

Supplementary table 3. Association between CES-D scores and risk of CVD death by sex (excluding deaths within 2 first years of observation)

	CES-D score	Czech Republic	Russia	Poland	3 countries (cluster)
		**HR (95%CI)**	**HR (95%CI)**	**HR (95%CI)**	**HR (95%CI)**
Men					
Age-adjusted	0-10	1.00	1.00	1.00	1.00
	11-15	1.07 (0.7-1.63)	1.3 (0.89-1.89)	1.42 (0.91-2.2)	1.24 (0.98-1.57)
	16-20	1.37 (0.75-2.51)	1.92 (1.19-3.08)	1.91 (1.15-3.19)	1.73 (1.28-2.33)
	21+	2.34 (1.39-3.95)	2.19 (1.38-3.5)	2.89 (1.85-4.51)	2.44 (1.86-3.2)
	p-value for trend	0.0035	0.0001	0.0000017	1.4E-11
Fully adjusted*	0-10	1.00	1.00	1.00	1.00
	11-15	0.7 (0.41-1.21)	1.2 (0.82-1.77)	1.11 (0.65-1.88)	1.02 (0.78-1.34)
	16-20	1.34 (0.68-2.67)	1.54 (0.94-2.53)	1.35 (0.72-2.55)	1.48 (1.06-2.07)
	21+	1.71 (0.88-3.33)	1.7 (1.04-2.79)	1.93 (1.1-3.38)	1.77 (1.29-2.43)
	p-value for trend	0.2	0.014	0.028	0.00018
Women					
Age-adjusted	0-10	1.00	1.00	1.00	1.00
	11-15	1.8 (0.97-3.33)	1.1 (0.59-2.04)	1.72 (0.95-3.09)	1.5 (1.06-2.14)
	16-20	1.5 (0.64-3.5)	1.54 (0.82-2.87)	1.67 (0.87-3.22)	1.61 (1.08-2.39)
	21+	2.33 (1.2-4.54)	1.77 (1-3.13)	1.96 (1.08-3.57)	2.02 (1.42-2.87)
	p-value for trend	0.012	0.036	0.022	0.000059
Fully adjusted*	0-10	1.00	1.00	1.00	1.00
	11-15	2.44 (0.97-6.1)	1.04 (0.55-1.95)	1.41 (0.66-3.02)	1.4 (0.92-2.14)
	16-20	2.34 (0.75-7.28)	1.41 (0.75-2.65)	2.17 (1.03-4.54)	1.83 (1.18-2.84)
	21+	1.65 (0.54-5.1)	1.24 (0.69-2.25)	1.27 (0.55-2.93)	1.44 (0.93-2.24)
	p-value for trend	0.26	0.41	0.32	0.053

* adjusted for age. education. marital status. occupational status. history of CVD. smoking. BMI. hypercholesterolemia. hypertension. physical activity. alkohol intake. diabetes

Supplementary table 4. Association between CES-D scores and risk of CVD death by sex in participants free of CVD at baseline

	CES-D score	Czech Republic	Russia	Poland	3 countries (cluster)
		**HR (95% CI)**	**HR (95% CI)**	**HR (95% CI)**	**HR (95% CI)**
Men					
Model 1	0-10	1.00	1.00	1.00	1.00
	11-15	1.46 (0.67-3.2)	1.42 (0.67-2.99)	1.86 (0.95-3.67)	1.62 (1.42-1.85)
	16-20	2.63 (1.13-6.09)	2.09 (0.99-4.42)	1.87 (0.87-4.03)	2.28 (1.89-2.75)
	21+	1.65 (0.65-4.17)	1.8 (0.84-3.89)	2.21 (1.09-4.49)	2.06 (1.87-2.27)
	p-value for trend	0.0015	<0.001	<0.001	<0.001
	1 sd	1.41 (1.18-1.69)	1.22 (1.02-1.45)	1.32 (1.1-1.58)	1.35 (1.27-1.43)
Model 2	0-10	1.00	1.00	1.00	1.00
	11-15	1.06 (0.6-1.87)	0.93 (0.58-1.49)	0.79 (0.42-1.51)	0.93 (0.83-1.05)
	16-20	1.14 (0.51-2.53)	1.65 (0.91-2.98)	1.74 (0.91-3.33)	1.52 (1.35-1.71)
	21+	2.97 (1.59-5.54)	1.96 (1.09-3.5)	2.39 (1.33-4.3)	2.28 (1.83-2.86)
	p-value for trend	0.0230	<0.001	<0.001	<0.001
	1 sd	1.23 (1.08-1.41)	1.23 (1.1-1.37)	1.34 (1.18-1.52)	1.29 (1.26-1.31)
Model 3	0-10	1.00	1.00	1.00	1.00
	11-15	2.26 (0.81-6.27)	1.47 (0.68-3.16)	1.69 (0.73-3.89)	1.82 (1.55-2.14)
	16-20	4.04 (1.38-11.82)	2.01 (0.93-4.35)	1.72 (0.71-4.18)	2.61 (1.83-3.71)
	21+	1.48 (0.39-5.69)	1.58 (0.71-3.5)	1.23 (0.46-3.34)	1.83 (1.47-2.29)
	p-value for trend	0.094	<0.001	0.0017	<0.001
	1 sd	1.42 (1.12-1.81)	1.1 (0.92-1.32)	1.17 (0.92-1.5)	1.29 (1.17-1.41)
Women					
Model 1	0-10	1.00	1.00	1.00	1.00
	11-15	1.46 (0.67-3.2)	1.42 (0.67-2.99)	1.86 (0.95-3.67)	1.62 (1.42-1.85)
	16-20	2.63 (1.13-6.09)	2.09 (0.99-4.42)	1.87 (0.87-4.03)	2.28 (1.89-2.75)
	21+	1.65 (0.65-4.17)	1.8 (0.84-3.89)	2.21 (1.09-4.49)	2.06 (1.87-2.27)
	p-value for trend	0.0027	0.0150	0.0160	<0.001
	1 sd	1.41 (1.18-1.69)	1.22 (1.02-1.45)	1.32 (1.1-1.58)	1.35 (1.27-1.43)
Model 2	0-10	1.00	1.00	1.00	1.00
	11-15	1.24 (0.55-2.82)	1.35 (0.64-2.85)	1.82 (0.92-3.59)	1.54 (1.3-1.82)
	16-20	2.47 (1.05-5.81)	1.99 (0.94-4.23)	1.69 (0.78-3.65)	2.13 (1.72-2.63)
	21+	1.29 (0.5-3.34)	1.53 (0.7-3.33)	1.94 (0.95-3.97)	1.79 (1.58-2.03)
	p-value for trend	0.0190	0.0910	0.0350	0.0000
	1 sd	1.32 (1.1-1.6)	1.14 (0.96-1.36)	1.29 (1.07-1.55)	1.29 (1.21-1.37)
Model 3	0-10	1.00	1.00	1.00	1.00
	11-15	2.26 (0.81-6.27)	1.47 (0.68-3.16)	1.69 (0.73-3.89)	1.82 (1.55-2.14)
	16-20	4.04 (1.38-11.82)	2.01 (0.93-4.35)	1.72 (0.71-4.18)	2.61 (1.83-3.71)
	21+	1.48 (0.39-5.69)	1.58 (0.71-3.5)	1.23 (0.46-3.34)	1.83 (1.47-2.29)
	p-value for trend	0.021	0.18	0.12	<0.001
	1 sd	1.42 (1.12-1.81)	1.1 (0.92-1.32)	1.17 (0.92-1.5)	1.29 (1.17-1.41)

Model 1 adjusted for age
Model 2 adjusted for age. education. marital status. occupational status
Model 3 adjusted for age. education. marital status. occupational status. history of cancer. smoking. BMI. hypercholesterolemia. hypertension. physical activity. alkohol intake

Supplementary table 5. Association between CES–D scores and risk of death from all causes by sex in participants free of CVD at baseline

	CES-D score	Czech Republic	Russia	Poland	3 countries (cluster)
		HR (95%CI)	HR (95%CI)	HR (95%CI)	HR (95%CI)
Men					
Model 1	0-10	1.00	1.00	1.00	1.00
	11-15	1.42 (1.06-1.89)	1.34 (1-1.8)	1.31 (0.98-1.76)	1.35 (1.31-1.4)
	16-20	1.7 (1.12-2.57)	1.53 (1-2.35)	1.98 (1.4-2.81)	1.74 (1.51-1.99)
	21+	2.63 (1.78-3.89)	1.91 (1.23-2.95)	2.55 (1.84-3.52)	2.31 (1.89-2.81)
	p-value for trend	<0.001	<0.001	<0.001	<0.001
	1 sd	1.26 (1.17-1.37)	1.21 (1.12-1.31)	1.3 (1.21-1.41)	1.27 (0-1.24)
Model 2	0-10	1.00	1.00	1.00	1.00
	11-15	1.4 (1.04-1.88)	1.29 (0.96-1.73)	1.17 (0.87-1.57)	1.28 (1.18-1.38)
	16-20	1.44 (0.94-2.21)	1.46 (0.95-2.25)	1.66 (1.17-2.36)	1.53 (1.4-1.67)
	21+	2.3 (1.54-3.43)	1.57 (1.01-2.45)	1.96 (1.4-2.75)	1.87 (1.53-2.29)
	p-value for trend	<0.001	<0.001	<0.001	<0.001
	1 sd	1.22 (1.12-1.32)	1.15 (1.05-1.25)	1.21 (1.12-1.31)	1.2 (1.18-1.22)
Model 3	0-10	1.00	1.00	1.00	1.00
	11-15	1.25 (0.87-1.79)	1.15 (0.85-1.56)	1.12 (0.79-1.6)	1.19 (1.12-1.26)
	16-20	1.71 (1.06-2.77)	1.37 (0.88-2.14)	1.65 (1.08-2.52)	1.53 (1.31-1.79)
	21+	2.24 (1.35-3.71)	1.24 (0.77-2)	1.82 (1.2-2.78)	1.66 (1.27-2.18)
	p-value for trend	0.001	0.0019	0.01	<0.001
	1 sd	1.19 (1.07-1.33)	1.14 (1.04-1.25)	1.14 (1.04-1.26)	1.17 (1.12-1.22)
Women					
Model 1	0-10	1.00	1.00	1.00	1.00
	11-15	1.62 (1.09-2.39)	1.61 (1.01-2.58)	1.34 (0.9-1.99)	1.52 (1.35-1.7)
	16-20	1.95 (1.2-3.17)	1.8 (1.07-3.03)	1.54 (1-2.38)	1.79 (1.55-2.06)
	21+	2.17 (1.41-3.35)	1.51 (0.88-2.57)	2.35 (1.63-3.39)	2.12 (1.74-2.59)
	p-value for trend	<0.001	0.017	<0.001	<0.001
	1 sd	1.41 (1.27-1.56)	1.19 (1.04-1.35)	1.34 (1.2-1.48)	1.33 (1.25-1.43)
Model 2	0-10	1.00	1.00	1.00	1.00
	11-15	1.48 (0.99-2.22)	1.59 (0.99-2.55)	1.27 (0.85-1.9)	1.45 (1.29-1.62)
	16-20	1.8 (1.1-2.97)	1.75 (1.04-2.94)	1.29 (0.83-2.01)	1.62 (1.33-1.99)
	21+	1.83 (1.17-2.87)	1.36 (0.79-2.34)	1.95 (1.35-2.83)	1.85 (1.6-2.13)
	p-value for trend	<0.001	0.077	<0.001	<0.001
	1 sd	1.35 (1.21-1.51)	1.14 (1-1.31)	1.25 (1.13-1.4)	1.27 (1.19-1.36)
Model 3	0-10	1.00	1.00	1.00	1.00
	11-15	1.33 (0.81-2.19)	1.66 (1.02-2.68)	1.08 (0.68-1.72)	1.37 (1.11-1.68)
	16-20	2.02 (1.13-3.6)	1.79 (1.05-3.03)	1.11 (0.66-1.85)	1.61 (1.17-2.22)
	21+	1.33 (0.73-2.42)	1.45 (0.84-2.51)	1.25 (0.78-2.02)	1.45 (1.3-1.63)
	p-value for trend	0.012	0.100	0.170	<0.001
	1 sd	1.3 (1.12-1.5)	1.11 (0.97-1.27)	1.1 (0.96-1.26)	1.2 (1.11-1.31)

Model 1 adjusted for age

Model 2 adjusted for age. education. marital status. occupational status

Model 3 adjusted for age. education. marital status. occupational status. history of cancer. smoking. BMI. hypercholesterolemia. hypertension. physical activity. alkohol intake

Supplementary figure 1. Distribution of CES-D scores by country and sex

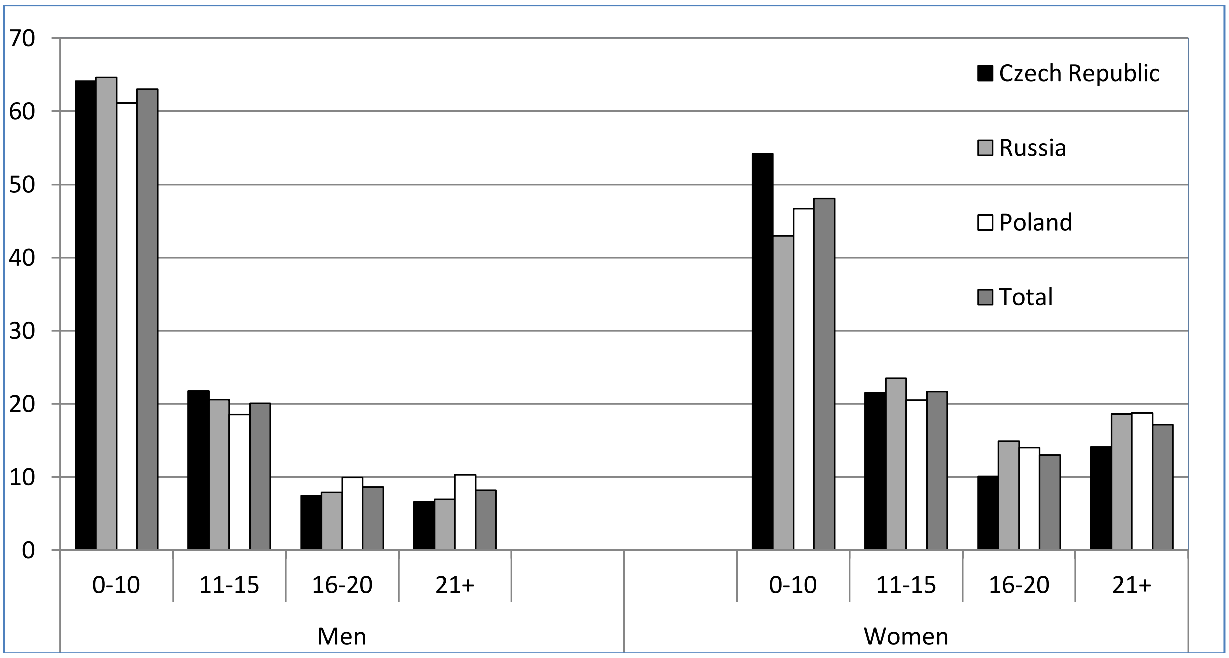

Supplement: Supplementary material [file suppl_TABLE_depression_mortality649493.pdf]
